# Vapor-Phase Anion Exchange in CH_3_NH_3_PbBr_3_ Perovskite Films: Continuous Bandgap Tuning and HI-Mediated Corrosion of ITO Substrates

**DOI:** 10.3390/mi17070797

**Published:** 2026-06-29

**Authors:** Honghong Xu, Yixian Zhang, Siyuan Liu, Feng Jiang

**Affiliations:** 1 School of Physics, Dalian University of Technology, Dalian 116024, China; xuhonghong@mail.dlut.edu.cn (H.X.); zhangyixian@mail.dlut.edu.cn (Y.Z.); 2 Leicester International Institute, Dalian University of Technology, Panjin 124221, China; 1263199057@mail.dlut.edu.cn; 3 School of General Education, Dalian University of Technology, Panjin 124221, China

**Keywords:** organic–inorganic hybrid perovskites, halide doping, vapor-phase anion exchange, bandgap tuning

## Abstract

CH_3_NH_3_PbBr_3_ crystalline films were prepared on ITO substrates using the spin-coating method, followed by a vapor-phase anion exchange process in a tube furnace using CH_3_NH_3_I to gradually replace the Br anions with I anions. By controlling the reaction time, the structural evolution and changes in optical properties were systematically investigated. X-ray diffraction patterns show that the I anions gradually replace the Br anions in the perovskite lattice as the reaction time increases, leading to lattice expansion and a shift in the diffraction peaks toward lower angles. Scanning electron microscopy reveals that the average grain size increases and the grain boundary reconstructs during the exchange process. Photoluminescence and UV–Vis absorption spectra show that the photoluminescence peak exhibits a continuous redshift, the absorption edge gradually shifts to longer wavelengths, and the optical bandgap decreases steadily toward the value of CH_3_NH_3_PbI_3_. A sharp increase in the resistivity of the ITO substrate was also observed. Control experiments confirm that this change is not due to thermal annealing but to the vapor-phase reaction between CH_3_NH_3_I and ITO. In the tube furnace, CH_3_NH_3_I is thermally decomposed into HI. HI not only promotes halide substitution but also diffuses to the ITO interface and etches In_2_O_3_ into insulating InI_3_, destroying the original conductive network. Therefore, this process is attributed to a HI-mediated multiphase reaction rather than a simple solid–vapor exchange. Overall, vapor-phase anion exchange provides an effective way to continuously tune the band structure, absorption range, and emission peak of hybrid perovskites, offering a controllable route for multicomponent perovskites and multiband optoelectronic devices. This work also emphasizes the potential chemical corrosion of bottom electrodes during the vapor-phase anion exchange process and suggests that protective measures such as barrier layers or corrosion-resistant electrodes should be considered.

## 1. Introduction

Organic–inorganic hybrid perovskites with the ABX_3_ structure exhibit direct bandgaps, high absorption coefficients, and long carrier diffusion lengths [1,2,3]. High-quality films and single crystals show low defect densities and efficient charge transport properties [4,5,6]. Their band structure and optical response can be tuned by compositional modification, enabling applications in solar cells [7], light-emitting diodes [8], lasers [9], and photodetectors [10].

Bandgap engineering can be achieved by adjusting the A-site cation, the B-site metal, or the X-site halide. Among these strategies, halide substitution at the X-site is the most convenient. Increasing Br^−^ content widens the bandgap and blueshifts the luminescence, while increasing I^−^ content narrows the bandgap and redshifts the emission [11,12,13]. This tunability makes halide perovskites attractive for applications that require precise control over absorption or emission wavelengths, such as tandem solar cells, tunable lasers, and full-color displays [11,12,13,14]. Post-synthesis anion exchange offers clear advantages over direct synthesis of mixed-halide perovskites. Direct synthesis requires re-optimization of preparation conditions for each composition and often suffers from inhomogeneity or phase segregation [15]. By contrast, anion exchange is performed on pre-synthesized perovskites with good morphology and crystallinity. This method decouples film formation from composition control, enabling wide-range bandgap tuning while preserving the original microstructure. Accordingly, anion exchange has become a widely used method for bandgap engineering in halide perovskites.

The majority of studies on anion exchange have been carried out using solution-based methods owing to the simple and mild conditions [16,17]. Compared with liquid-phase treatment, vapor-phase anion exchange has attracted extensive attention because of its advantages, including more uniform reaction and less damage to the films. Although vapor-phase exchange avoids solvent-induced damage and preserves film structure better [18,19], the detailed mechanism of halide conversion under vapor conditions is still under exploration. Kim et al. achieved in situ Br^−^/I^−^ exchange in perovskite films through a vapor-phase halide exchange method and systematically investigated the exchange kinetics process [20]. The study by Barrit et al. reported that through a gas–solid interfacial sequential halide exchange/vapor-assisted reaction approach, a stepwise transformation from single-halide (e.g., I^−^) to mixed-halide (I^−^/Br^−^) lead-based perovskite thin films was achieved, resulting in CH_3_NH_3_Pb(I_1−x_Br_x_)_3_ films with precisely tunable bandgaps [21]. Karimata et al. further pointed out that during the halide exchange process, perovskite crystals could achieve compositional regulation and bandgap tuning while maintaining their original morphology [22]. These previous works on vapor-phase anion exchange, including the study by Zhang et al. on the CH_3_NH_3_PbI_3_/CH_3_NH_3_Br system, mainly focused on replacing I^−^ with Br^−^ using CH_3_NH_3_Br vapor at controlled evaporation rates [23]. In that system, Br^−^ substitution is more favorable because the activation energy for vacancy migration of Br^−^ along the (100) direction (0.258 eV) is lower than that of I^−^ (0.329 eV). Zhang et al. described the process as a direct solid–gas reaction: CH_3_NH_3_PbI_3_ + xCH_3_NH_3_Br → CH_3_NH_3_PbI_3−x_Br_x_ + xCH_3_NH_3_I and proposed a logarithmic relationship between Br content and reaction time, attributing the kinetic behavior to the slow release of CH_3_NH_3_Br vapor. Whether vapor-phase anion exchange using CH_3_NH_3_I in a tube furnace can effectively substitute Br^−^ with I^−^ in CH_3_NH_3_PbBr_3_ films, and the underlying mechanism of such an exchange process, remains to be elucidated.

In this work, vapor-phase anion exchange of CH_3_NH_3_PbBr_3_ crystalline films with CH_3_NH_3_I was carried out in a tube furnace. The characterizations of structural and optical properties show a gradual conversion from CH_3_NH_3_PbBr_3_ to CH_3_NH_3_PbI_3_. The photoluminescence (PL) peak shifts continuously from ~550 nm to ~790 nm, the absorption edge moves from ~560 nm to ~810 nm, and the optical bandgap decreases from 2.23 eV to 1.55 eV. A significant increase in the resistivity of the ITO substrate was observed after the anion exchange. Based on these systematic measurements, the anion exchange mechanism was discussed. The results demonstrate that vapor-phase anion exchange enables continuous bandgap tuning over a broad spectral range, providing a simple and effective route for composition engineering in hybrid perovskites. In addition, this work provides an experimental basis for substrate selection in vapor–solid anion exchange or thermal synthesis of perovskite based on CH_3_NH_3_I and reveals that ITO is susceptible to etching by HI.

## 2. Materials and Methods

**Figure 1:**
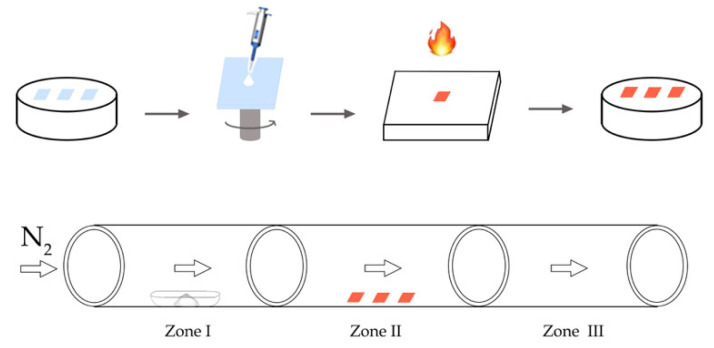
The schematic diagram of the preparation process and anion exchange for the CH_3_NH_3_PbBr_3_ perovskite films.

The ITO substrates were sequentially cleaned in deionized water, absolute ethanol and acetone, each for 15 min in an ultrasonic bath. The substrates were then wiped repeatedly with absolute ethanol until the surface was thoroughly cleaned. Afterward, the substrates were dried in a vacuum oven. A precursor solution was prepared by dissolving 482.2 mg of PbBr_2_ and 147.1 mg of CH_3_NH_3_Br (the molar ratio of PbBr_2_ to CH_3_NH_3_Br is 1:1) in 1 mL of N,N-dimethylformamide (DMF). The mixture was stirred at room temperature for 6 h to obtain a clear solution. CH_3_NH_3_PbBr_3_ crystalline films were fabricated via the spin-coating method, as shown in Figure 1. 20 μL of the perovskite precursor solution was dropped onto the substrate fixed on the spin coater. The substrate was then spun at 500 rpm for 5 s and then at 1000 rpm for 20 s. After spin-coating, the substrates were annealed on a hot plate at 120 °C for 45 min to obtain orange CH_3_NH_3_PbBr_3_ perovskite films. For the vapor-phase anion exchange process, 40 mg of CH_3_NH_3_I powder was placed at the center of the first heating zone in an SK-1200 three-zone vacuum tube furnace, which was set to 190 °C. The as-prepared CH_3_NH_3_PbBr_3_ films were placed at the center of the second zone, maintained at 160 °C, while the third zone was set to the same temperature to ensure thermal stability. Different reaction times were employed to achieve different extents of anion exchange, with a nitrogen carrier gas (99.999% purity) flow of 100 sccm. After the reaction, the samples were cooled naturally to room temperature inside the furnace (approximately 1 °C/min).

The surface morphology of the perovskite films was characterized using a field-emission scanning electron microscope (SEM 450, FEI, Thermo Fisher, Waltham, MA, USA). The crystal structure and phase composition of the perovskite films were analyzed by X-ray diffraction (XRD, Shimadzu, Kyoto, Japan) with Cu Kα radiation (λ = 1.54056 Å), recorded over a 2*θ* range of 10–70° at a scanning rate of 4°/min. PL spectra were measured using a steady-state fluorescence spectrometer (Hitachi F-7000, Hitachi High-Technologies Corporation, Tokyo, Japan). The absorption edge of the perovskite films was determined using a UV–Vis–NIR spectrophotometer (Lambda 950, PerkinElmer Instruments, Buckinghamshire, UK). Keithley 2460 (Keithley Instruments, LLC, Cleveland, OH, USA) was used to record the current–voltage (I–V) curves of the ITO substrates after 5 h of anion exchange process. The elemental composition of the ITO substrates before and after the anion exchange process was analyzed using X-ray photoelectron spectrometer (XPS, ESCALAB 250Xi, Thermo Fisher Scientific, Waltham, MA, USA). In addition, the elemental composition and relative content of the perovskite films were analyzed by energy-dispersive X-ray spectroscopy (EDS) attached to the SEM.

## 3. Results and Discussion

**Figure 2:**
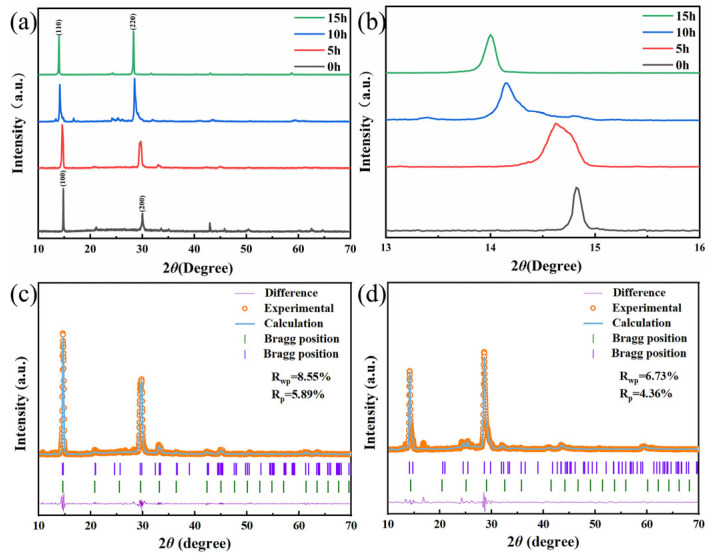
(**a**) XRD patterns of the CH_3_NH_3_PbI_3−x_Br_x_ films after different reaction times. (**b**) Enlarged view of the XRD peak at 13–16°. Rietveld refinement profiles of the CH_3_NH_3_PbBr_3_ film after 5 h (**c**) and 10 h (**d**) of anion exchange.

Figure 2a shows the XRD patterns of the CH_3_NH_3_PbI_3−x_Br_x_ films after different reaction times. As the exchange time increases, all diffraction peaks gradually shift to lower angles, indicating the incorporation of I^−^ into the lattice. CH_3_NH_3_PbI_3_ has a tetragonal structure [24], whereas CH_3_NH_3_PbBr_3_ belongs to the cubic phase [25]. At the reaction time of 15 h, the crystal structure has transformed from the cubic phase to the tetragonal phase. Figure 2b shows the enlarged XRD patterns around 15° of the CH_3_NH_3_PbI_3−x_Br_x_ films after different reaction times. For the 5 h and 10 h samples, both peaks display poor symmetry and marked broadening, indicating the possible coexistence of the two cubic and tetragonal phases [26]. At the exchange time of 15 h, the diffraction peak exhibits high symmetry. These results show that iodine substitution can effectively tune the crystal structure of the perovskite films. Rietveld refinement of the XRD patterns for the 5 h and 10 h samples was carried out using the GSAS-II package, with cubic CH_3_NH_3_PbBr_3_ (space group Pm-3m) and tetragonal CH_3_NH_3_PbI_3_ (space group I4/mcm) as starting models. The refinement confirms the coexistence of a cubic phase and a tetragonal phase in both samples, as shown in Figure 2c,d. The refined lattice parameters show systematic changes. The cubic lattice constant a expands from 6.038 Å to 6.152 Å, while the tetragonal c axis lengthens from 12.098 Å to 12.495 Å. The cubic lattice parameter is significantly larger than that of pure CH_3_NH_3_PbBr_3_ (~5.93 Å), indicating substantial I^−^ incorporation into the cubic phase, while the tetragonal c axis remains shorter than that of pure CH_3_NH_3_PbI_3_ (~12.62 Å), suggesting considerable Br^−^ retention in the tetragonal phase. Thus, both phases are described as mixed-halide solid solutions differing in their relative I/Br enrichment, rather than as end-member bromide and iodide phases. Quantitative phase analysis gives a cubic fraction of approximately 68 wt% and a tetragonal fraction of approximately 32 wt% for the 5 h sample. After 10 h, the tetragonal fraction increases to approximately 75 wt% and the cubic fraction decreases to approximately 25 wt%. The refinement yields reliability factors of R_wp_ = 8.55% and R_p_ = 5.89%, indicating acceptable agreement between the calculated and observed patterns. The refined weight fractions (~68 wt% cubic and ~32 wt% tetragonal at 5 h; ~25 wt% cubic and ~75 wt% tetragonal at 10 h) represent the relative abundances of the two crystallographic phases, not the halide distribution between them. The estimated standard deviations for the refined parameters are provided in Table S1 (Supplementary Information).

**Figure 3:**
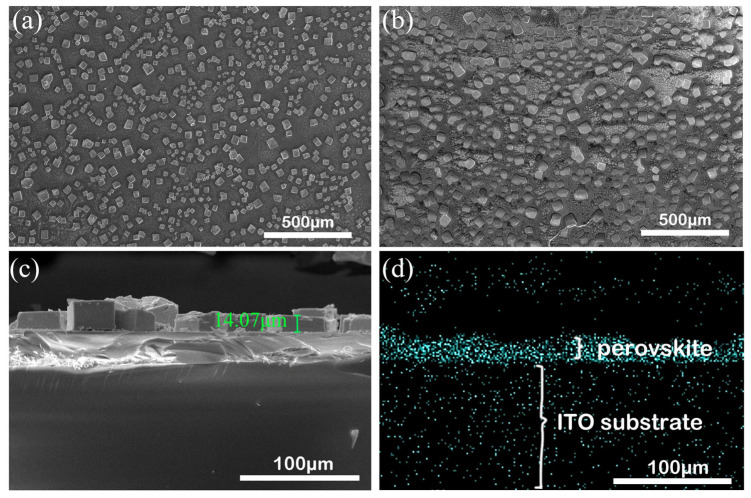
SEM surface images of the CH_3_NH_3_PbBr_3_ film before (**a**) and after (**b**) 15 h of anion exchange. (**c**) The cross-sectional SEM images of a representative CH_3_NH_3_PbBr_3_ grain on the ITO substrate. (**d**) The cross-sectional elemental mapping of I on the 10 h sample.

Figure 3a presents the SEM image of the CH_3_NH_3_PbBr_3_ crystalline film before anion exchange. The surface is covered with uniformly distributed, well-defined block-like crystals that exhibit a cubic morphology. Grain boundaries are relatively clear and sharp, indicating high crystalline quality and a low surface defect density. After 15 h of treatment, the surface morphology undergoes a transformation, as shown in Figure 3b. The grains become larger, and the originally sharp crystal edges turn somewhat passivated. This morphological evolution is due to the ion migration and lattice reconstruction during the exchange process [27]. The thickness of the crystalline film was measured by cross-sectional SEM, and the results indicate that the overall thickness of the film falls within the range of 14–25 μm, as shown in Figure 3c. Previous studies have shown that in halide perovskite systems, halide anions possess low migration barriers and are prone to diffusion under thermal driving or concentration gradients [28]. During the anion exchange reaction, the ion exchange behavior of the material is predominantly governed by a surface-dominated mechanism, which is usually accompanied by localized dissolution–recrystallization. Specifically, partial dissolution takes place on crystal surfaces, followed by re-nucleation and crystal growth induced by ion redistribution. This process ultimately results in enlarged grain size and reconstructed grain boundaries [29].

As the reaction time increases from 1 h to 15 h, the I/(I + Br) atomic ratio rises from 0.004 to 0.887, meaning that the composition parameter x in CH_3_NH_3_PbI_3−x_Br_x_ drops from about 2.98 to 0.34 (Table 1). This shows a continuous and controllable anion exchange process. Iodine incorporation is very slow in the first few hours. The EDS spectrum and full composition table of the CH_3_NH_3_PbI_3−x_Br_x_ films measured at different anion exchange times are provided in Table S2 (Supplementary Information). At 2–3 h, the I/(I + Br) ratio is below 0.05, then increases gradually, and after 10 h jumps to 0.887 at 15 h. This differs from the logarithmic exchange reported by Zhang et al. for Br replacing I in CH_3_NH_3_PbI_3_ films [23]. The as-prepared CH_3_NH_3_PbBr_3_ film is a kind of discontinuous micrometer-scale crystalline film with few grain boundaries (Figure 3a,c). The larger I^−^ cannot easily enter such a compact structure. Therefore, the early stage is not limited by chemistry or vapor supply but by the difficulty of getting I^−^ into a dense film with few diffusion paths. To find the rate-limiting step, cross-sectional iodine mapping was performed on the 10 h sample (Figure 3d). The results show that iodine is distributed uniformly across the film thickness. This uniform interior distribution indicates that once I^−^ manages to get through the top surface, it diffuses rapidly through the perovskite lattice. Thus, the internal diffusion is not the bottleneck. What really limits the early-stage exchange is the entry of I^−^ into the dense crystalline network. As a certain amount of I^−^ eventually does get in, it triggers grain growth and grain boundary reconstruction, as shown in Figure 3b. These microstructural changes create new diffusion pathways that can accommodate the larger I^−^ ions, making the subsequent exchange much faster. This explains why the iodine fraction stays low before 10 h but then increases sharply afterward.

**Figure 4:**
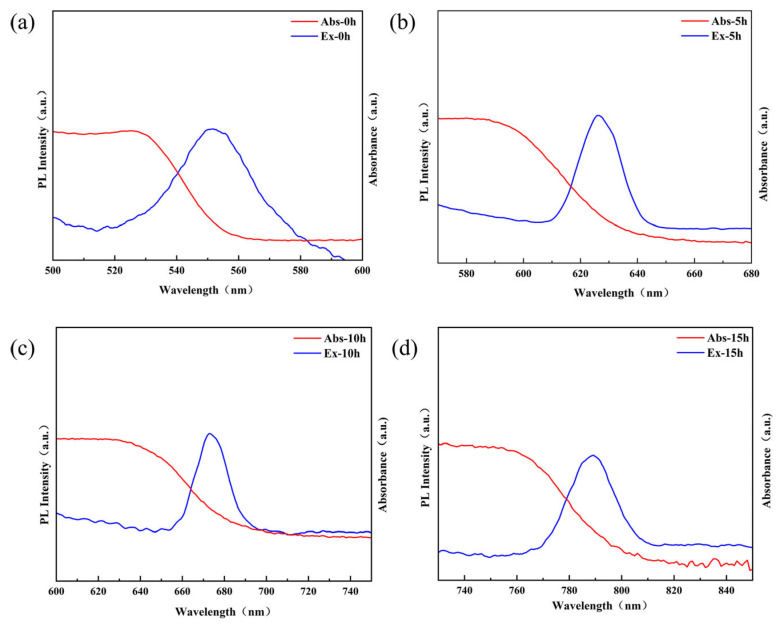
Vis–NIR absorption and PL spectra of the CH_3_NH_3_PbBr_3_ films after (**a**) 0h, (**b**) 5h, (**c**) 10h, and (**d**) 15 h of anion exchange.

Figure 4 shows the PL and UV–Vis–NIR absorption spectra of the CH_3_NH_3_PbI_3−x_Br_x_ films at different reaction times. As shown in Figure 4a, the pristine CH_3_NH_3_PbBr_3_ film exhibits a PL peak at approximately 550 nm. After a 5 h reaction with CH_3_NH_3_I, the PL peak redshifts to 625 nm (Figure 4b), indicating that the incorporation of a small amount of I^−^ into the lattice has already changed the band structure of the material. With further prolongation of reaction time, the PL peak undergoes a continuous redshift. As displayed in Figure 4c, the emission peak is located at around 675 nm after 10 h of reaction, whereas it shifts to 790 nm at 15 h (Figure 4d), which is close to the characteristic emission wavelength of CH_3_NH_3_PbI_3_. The pristine CH_3_NH_3_PbBr_3_ film exhibits an absorption edge located at approximately 560 nm. With prolonged reaction time, the absorption edge gradually moves toward longer wavelengths, which is consistent with the redshift trend of PL emission. Specifically, the absorption edge shifts to ~650 nm after 5 h reaction, reaches ~690 nm at 10 h, and further redshifts to around 810 nm after 15 h. From the perspective of electronic band structure, the substitution of Br^−^ by I^−^ alters energy levels of the halide p-orbital and the Pb-X orbital coupling, resulting in an upward shift in the valence band maximum and a reduction in the bandgap [30]. Therefore, with increasing I content, the absorption edge exhibits a progressive redshift, extending the spectral response from the short-wavelength region toward longer wavelengths within the visible spectrum. These results demonstrate that vapor-phase anion exchange using CH_3_NH_3_I can not only regulate the chemical composition of the CH_3_NH_3_PbBr_3_ film but also achieve effective modulation of their optical absorption and emission characteristics.

**Figure 5:**
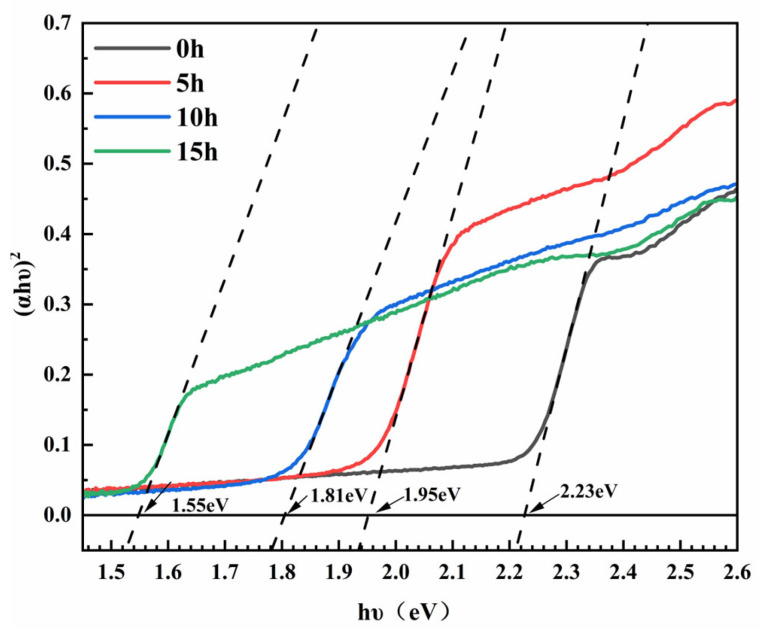
(αhν)^2^ vs. hν of optical absorption spectra of the CH_3_NH_3_PbI_3−x_Br_x_ films after different reaction times.

The optical bandgap determines the spectral response range of the material, and a narrower band gap is typically associated with improved light absorption in the long-wavelength region. In this work, the optical bandgaps of the CH_3_NH_3_PbI_3−x_Br_x_ films were estimated using the Tauc relation for direct-bandgap semiconductors:

(1) $$\begin{array}{c} {\left(\alpha h\nu \right)}^{2}=c\left(h\nu -{Ε}_{g}\right) \end{array}$$

where *α* is the absorption coefficient, *hν* is the photon energy, *c* is a constant, and *E_g_* is the optical bandgap [31]. As shown in Figure 5, the Tauc plot indicates that the bandgap of the pristine CH_3_NH_3_PbBr_3_ film is approximately 2.23 eV. After 5 h of reaction, the bandgap declines to 1.95 eV, and further decreases to 1.81 eV at 10 h, finally reaching about 1.55 eV after 15 h. This trend indicates a gradual transition from wide-bandgap to narrow-bandgap characteristics. Previous studies have confirmed that changes in the I/Br ratio significantly affect the absorption edge and emission peak [32,33]. The bandgap evolution observed in this work is consistent with these reports. The narrowing of the optical bandgap is attributed to lattice expansion and electronic structure reconstruction induced by halide substitution. Lindblad et al. revealed that the valence band maximum in CH_3_NH_3_PbX_3_ is strongly influenced by halide energy levels; therefore, changing the halide species alters the band structure [30]. With the gradual substitution of Br^−^ by I^−^, the valence band maximum shifts upward, accompanied by continuous bandgap narrowing. This phenomenon induces the redshift of both absorption edge and PL emission peak. The measured bandgap values are compared with the composition-dependent bandgap relationship reported by Zhang et al. [23], which is described by the following equation:

(2) $$E_{g}(x)=E_{g}(0)+\frac{\left[E_{g}(3)-E_{g}(0)-b\right]}{3}x+\frac{b}{9}x^{2}$$

where *E_g_*(*x*) refers to the bandgap of CH_3_NH_3_PbI_3−x_Br_x_. *E_g_*(0) and *E_g_*(3) correspond to the bandgaps of CH_3_NH_3_PbI_3_ (1.58 eV) and CH_3_NH_3_PbBr_3_ (2.23 eV). The bending parameter b (0.90 eV) is related to fluctuation degrees in the crystal field of CH_3_NH_3_PbI_3−x_Br_x_.The experimental data points fall almost exactly on the same curves predicted by Equation (1), indicating excellent agreement with the established bandgap–composition relationship.

**Figure 6:**
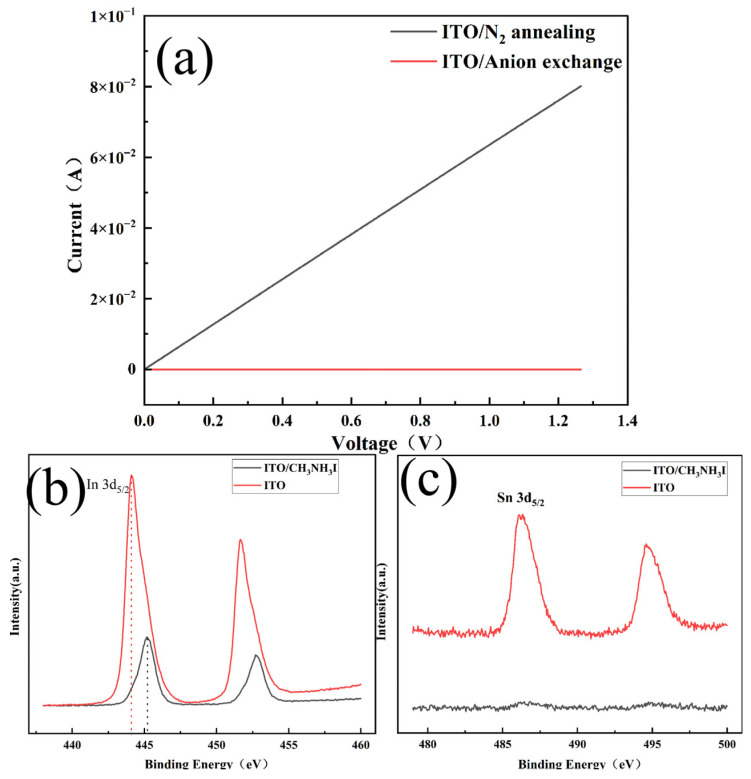
(**a**) The I–V curves of ITO substrates under different atmospheres. The In 3d (**b**) and Sn 3d (**c**) XPS spectra of the ITO substrates before and after 5 h of anion exchange.

In addition, a significant increase in the resistivity of the ITO substrate was observed after 5 h of anion exchange, as shown in Figure 6a. In contrast, a bare ITO substrate annealed under identical conditions (same temperature, N_2_ atmosphere, without CH_3_NH_3_I, 5 h) exhibited negligible change in conductivity, indicating that the resistivity rise is not attributable solely to thermal annealing but is directly associated with the vapor-phase reaction involving CH_3_NH_3_I. If the anion exchange proceeded as a simple solid-state reaction, i.e., CH_3_NH_3_PbBr_3_ + CH_3_NH_3_I → CH_3_NH_3_PbI_3_ + CH_3_NH_3_Br, none of the products would appreciably interact with ITO. Consequently, the actual reaction pathway must be more complex. It is proposed that CH_3_NH_3_I first undergoes thermal decomposition into methylamine (CH_3_NH_2_) and hydrogen iodide (HI) in the tube furnace. As a kind of Brønsted acid, HI is highly reactive. On one hand, it reacts with CH_3_NH_3_PbBr_3_ to yield CH_3_NH_3_PbI_3_ and HBr, thereby converting the perovskite layer from bromide to iodide. On the other hand, HI can diffuse to the ITO interface and chemically etch its primary component, In_2_O_3_ and SnO_2_, according to the reaction: In_2_O_3_ + 6HI → 2InI_3_+ 3H_2_O, SnO_2_ + 4HI → SnI_4_ + 2H_2_O. Figure 6b shows the In 3d XPS spectra of the ITO substrates before and after 5 h of anion exchange. XPS analysis corroborates the proposed etching mechanism. Before anion exchange, the In 3d_5_/_2_ peak of the ITO substrate is located at 444.2 eV, which is consistent with the characteristic binding energy of In_2_O_3_ [34]. After the vapor-phase anion exchange treatment with CH_3_NH_3_I, the In 3d_5/2_ peak shifts significantly to 445.2 eV, a value that is characteristic of InI_3_ [34]. This shift of about 1.0 eV toward higher binding energy corresponds to a decrease in electron density at the In sites upon transition from an oxide to an iodide coordination environment. XPS analysis further reveals the complete disappearance of the Sn 3d peaks from the ITO substrate after 5 h of anion exchange (Figure 6c). This observation indicates that SnO_2_, the tin-containing component of ITO, also reacts with HI generated from the thermal decomposition of CH_3_NH_3_I. The complete loss of the Sn signal is likely attributable to SnI_4_ volatilization, based on its low melting point (144 °C) relative to the reaction temperature (160 °C) and the N_2_ flow. Direct evidence is absent, as downstream trapping on activated carbon failed to detect Sn, presumably owing to the negligible total Sn mass in the ITO layer (~120 nm, ~10 at%). Accordingly, volatilization is considered the most reasonable hypothesis, though not conclusively proven. The XPS data provides direct experimental evidence for the proposed HI-mediated multiphase reaction pathway. HI diffuses to the ITO interface and reacts with In_2_O_3_ and SnO_2_, disrupting the conductive network, generating high-resistivity InI_3_, and leading to a sharp increase in substrate resistivity. Therefore, the vapor-phase anion exchange does not occur via a simple solid-state exchange but rather through a HI-mediated multiphase reaction pathway: CH_3_NH_3_I → CH_3_NH_2_ + HI, CH_3_NH_3_PbBr_3_ + HI → CH_3_NH_3_PbI_3_ + HBr.

The proposed mechanism also reveals that, during vapor-phase anion exchange for compositional tuning of perovskites, decomposition byproducts in the reaction atmosphere can cause unintended chemical corrosion of underlying functional layers or electrodes. Therefore, in device integration or patterning processes, attention should be paid to such HI-mediated reactions that compromise the stability of the substrate and electrodes. Mitigation strategies include the use of protective layers, optimization of reaction temperatures, or the adoption of corrosion-resistant electrode materials.

## 4. Conclusions

In summary, vapor-phase anion exchange using CH_3_NH_3_I successfully converts CH_3_NH_3_PbBr_3_ crystalline films into CH_3_NH_3_PbI_3−x_Br_x_ with continuously tunable composition. Increasing the reaction time progressively incorporates I^−^ into the lattice, leading to a redshift in the PL peak, a redshift in the absorption edge, and a decrease in the optical bandgap from 2.23 eV to 1.55 eV. Structural transformation from cubic to tetragonal phase accompanies grain growth and grain boundary reconstruction. Notably, a marked increase in ITO substrate resistivity is observed after the exchange. Control experiments and XPS analysis reveal that thermally decomposed HI from CH_3_NH_3_I etches In_2_O_3_ into insulating InI_3_. Thus, the process proceeds via a HI-mediated multiphase reaction rather than a simple solid–vapor exchange. This finding underscores the potential chemical corrosion of bottom electrodes during vapor-phase anion exchange. Mitigation strategies such as protective layers or corrosion-resistant electrodes are recommended for device integration.

## Figures and Tables

**Table 1:** Atomic percentages of I and Br, I/(I + Br) ratio, and corresponding x values in CH_3_NH_3_PbI_3−x_Br_x_ as a function of reaction time (measured by EDS).

Reaction Time	I Atomic %	Br Atomic %	I/(I + Br)	The Value of x for CH_3_NH_3_PbI_3−x_Br_x_
1 h	0.09	20.09	0.004	2.98
2 h	0.78	21.56	0.035	2.90
3 h	1.09	20.65	0.050	2.85
4 h	3.15	16.73	0.158	2.52
5 h	4.7	18.18	0.205	2.38
7.5 h	5.46	17.93	0.233	2.30
10 h	7.81	14.72	0.347	1.96
12.5 h	18.37	6.44	0.740	0.78
15 h	11.68	1.49	0.887	0.34

## Data Availability

The original contributions presented in this study are included in the article. Further inquiries can be directed to the corresponding author.

## Supplementary Materials

The following supporting information can be downloaded at: https://www.mdpi.com/article/10.3390/mi17070797/s1, Table S1: Rietveld refinement results: lattice parameters and quantitative phase fractions (wt%) for the CH_3_NH_3_PbI_3−x_Br_x_ films at selected anion-exchange durations. Estimated standard deviations are given in parentheses; Table S2: The EDS spectrum and full composition table of the CH_3_NH_3_PbI_3−x_Br_x_ films measured at different anion-exchange times.

## References

[1] Snaith H.J. (2013). Perovskites: The emergence of a new era for low-cost, high-efficiency solar cells. J. Phys. Chem. Lett..

[2] Zhang W., Eperon G.E., Snaith H.J. (2016). Metal halide perovskites for energy applications. Nat. Energy.

[3] De Wolf S., Holovsky J., Moon S.-J., Löper P., Niesen B., Ledinsky M., Haug F.-J., Yum J.-H., Ballif C. (2014). Organometallic Halide Perovskites: Sharp Optical Absorption Edge and Its Relation to Photovoltaic Performance. J. Phys. Chem. Lett..

[4] Stranks S.D., Eperon G.E., Grancini G., Menelaou C., Alcocer M.J.P., Leijtens T., Herz L.M., Petrozza A., Snaith H.J. (2013). Electron-Hole Diffusion Lengths Exceeding 1 Micrometer in an Organometal Trihalide Perovskite Absorber. Science.

[5] Xing G., Mathews N., Sun S., Lim S.S., Lam Y.M., Grätzel M., Mhaisalkar S., Sum T.C. (2013). Long-Range Balanced Electron- and Hole-Transport Lengths in Organic-Inorganic CH_3_NH_3_PbI_3_. Science.

[6] Shi D., Adinolfi V., Comin R., Yuan M., Alarousu E., Buin A., Chen Y., Hoogland S., Rothenberger A., Katsiev K. (2015). Low trap-state density and long carrier diffusion in organolead trihalide perovskite single crystals. Science.

[7] Seok S.I., Grätzel M., Park N.-G. (2018). Methodologies toward Highly Efficient Perovskite Solar Cells. Small.

[8] Lin K., Xing J., Quan L.N., García de Arquer F.P., Gong X., Lu J., Xie L., Zhao W., Zhang D., Yan C. (2018). Perovskite light-emitting diodes with external quantum efficiency exceeding 20 per cent. Nature.

[9] Chen J., Du W., Shi J., Li M., Wang Y., Zhang Q., Liu X. (2020). Perovskite quantum dot lasers. InfoMat.

[10] Li Y., Shi Z., Lei L., Ma Z., Zhang F., Li S., Wu D., Xu T., Li X., Shan C. (2018). Controllable Vapor-Phase Growth of Inorganic Perovskite Microwire Networks for High-Efficiency and Temperature-Stable Photodetectors. ACS Photon..

[11] Xing G., Mathews N., Lim S.S., Yantara N., Liu X., Sabba D., Grätzel M., Mhaisalkar S., Sum T.C. (2014). Low-temperature solution-processed wavelength-tunable perovskites for lasing. Nat. Mater..

[12] Noh J.H., Im S.H., Heo J.H., Mandal T.N., Seok S.I. (2013). Chemical Management for Colorful, Efficient, and Stable Inorganic-Organic Hybrid Nanostructured Solar Cells. Nano Lett..

[13] Miah M.H., Khandaker M.U., Rahman M.B., Nur-E-Alam M., Islam M.A. (2024). Band gap tuning of perovskite solar cells for enhancing the efficiency and stability: Issues and prospects. RSC Adv..

[14] Kim Y.-H., Cho H., Lee T.-W. (2016). Metal halide perovskite light emitters. Proc. Natl. Acad. Sci. USA.

[15] Mosconi E., Amat A., Nazeeruddin M.K., Grätzel M., De Angelis F. (2013). First-Principles Modeling of Mixed Halide Organometal Perovskites for Photovoltaic Applications. J. Phys. Chem. C.

[16] Huang H., Raith J., Kershaw S.V., Kalytchuk S., Tomanec O., Jing L., Susha A.S., Zbořil R., Rogach A.L. (2017). Growth mechanism of strongly emitting CH_3_NH_3_PbBr_3_ perovskite nanocrystals with a tunable bandgap. Nat. Commun..

[17] Karimata I., Kobori Y., Tachikawa T. (2017). Direct Observation of Charge Collection at Nanometer-Scale Iodide-Rich Perovskites during Halide Exchange Reaction on CH_3_NH_3_PbBr_3_. J. Phys. Chem. Lett..

[18] Pellet N., Teuscher J., Maier J., Gratzel M. (2015). Transforming Hybrid Organic Inorganic Perovskites by Rapid Halide Exchange. Chem. Mater..

[19] Zhang K., Shen Y., Cao L.X., Su Z.H., Hu X.M., Feng S.C., Wang B.F., Xie F.M., Li H.Z., Gao X. (2024). Nondestructive halide exchange via SN2-like mechanism for efficient blue perovskite light-emitting diodes. Nat. Commun..

[20] Kim G., An S., Hyeong S.-K., Lee S.-K., Kim M., Shin N. (2021). In Situ Vapor-Phase Halide Exchange of Patterned Perovskite Thin Films. Small.

[21] Barrit D., Zhang Y., Yang T., Tang M.-C., Li R., Smilgies D.-M., Liu S., Anthopoulos T.D., Amassian A., Zhao K. (2021). Sequential Formation of Tunable-Bandgap Mixed-Halide Lead-Based Perovskites: In Situ Investigation and Photovoltaic Devices. Sol. RRL.

[22] Karimata I., Tachikawa T. (2021). In Situ Exploration of the Structural Transition during Morphology- and Efficiency-Conserving Halide Exchange on a Single Perovskite Nanocrystal. Angew. Chem. Int. Ed. Engl..

[23] Zhang Z., Wei H., Manohari A.G., You D., Wang R., Li Z., Liu W., Chen J., Zhu Y., Shi Z. (2021). In Situ and Quantitative Vapor/Solid Anion Exchange for Composition Regulation and Optical Properties of Perovskites. Adv. Opt. Mater..

[24] Hermes I.M., Bretschneider S.A., Bergmann V.W., Li D., Klasen A., Mars J., Tremel W., Laquai F., Butt H.-J., Mezger M. (2016). Ferroelastic fingerprints in methylammonium lead iodide perovskite. J. Phys. Chem. C.

[25] Baikie T., Barrow N.S., Fang Y., Keenan P.J., Slater P.R., Piltz R.O., Gutmann M., Mhaisalkar S.G., White T.J. (2015). A combined single crystal neutron/X-ray diffraction and solid-state nuclear magnetic resonance study of the hybrid perovskites CH_3_NH_3_PbX_3_ (X = I, Br and Cl). J. Mater. Chem. A.

[26] Sadhanala A., Deschler F., Thomas T.H., Dutton S.E., Goedel K.C., Hanusch F.C., Lai M.L., Steiner U., Bein T., Docampo P. (2014). Preparation of single-phase films of CH_3_NH_3_Pb(I_1−x_Br_x_)_3_ with sharp optical band edges. J. Phys. Chem. Lett..

[27] Scheidt R.A., Kamat P.V. (2019). Temperature-driven anion migration in gradient halide perovskites. J. Chem. Phys..

[28] Thiesbrummel J., Milić J.V., Deibel C., Garnett E.C., Tao S., Kirchartz T., Guerrero A., Cameron P., Tress W., Saiful Islam M. (2026). Ion migration in perovskite solar cells. Nat. Rev. Chem..

[29] Zhao H., Wang Y., Wang C., Bandela A.K., Thumu U. (2023). Dissolution-dictated recrystallization in cesium lead halide perovskites and size engineering in δ-CsPbI_3_ nanostructures. Cryst. Growth Des..

[30] Lindblad R., Jena N.K., Philippe B., Oscarsson J., Bi D., Lindblad A., Mandal S., Pal B., Sarma D.D., Karis O. (2015). Electronic structure of CH_3_NH_3_PbX_3_ perovskites: Dependence on the halide moiety. J. Phys. Chem. C.

[31] Samanta D., Chaudhary S.P., Ghosh B., Bhattacharyya S., Shukla G., Mukherjee G.D. (2022). Pressure-induced emission enhancement and bandgap narrowing: Experimental investigations and first-principles theoretical simulations on the model halide perovskite Cs_3_Sb_2_Br_9_. Phys. Rev. B.

[32] D’Innocenzo V., Srimath Kandada A.R., De Bastiani M., Gandini M., Petrozza A. (2014). Tuning the light emission properties by band gap engineering in hybrid lead halide perovskite. J. Am. Chem. Soc..

[33] Xiao Z., Zhao L., Tran N.L., Lin Y.L., Silver S.H., Kerner R.A., Yao N., Kahn A., Scholes G.D., Rand B.P. (2017). Mixed-halide perovskites with stabilized bandgaps. Nano Lett..

[34] Henderson J.D., Pearson L., Nie H.Y., Biesinger M.C. (2024). X-ray photoelectron spectroscopy analysis of indium and indium-containing compounds. Surf. Interface Anal..

