# Supplementary material for: Vapor-Phase Anion Exchange in CH3NH3PbBr3 Perovskite Films: Continuous Bandgap Tuning and HI-Mediated Corrosion of ITO Substrates
Source: Micromachines (Basel). 2026 Jun 29;17(7):797. doi: 10.3390/mi17070797 (PMC13413708; doi:10.3390/mi17070797)
Supplement: Supplementary file 1 [file micromachines-17-00797-s001.zip › micromachines-4363176-supplementary.pdf]

Supplementary Materials

Table S1. Rietveld refinement results: lattice parameters and quantitative phase fractions (wt%) for the $CH_3NH_3PbI_{3-x}Br_x$ films at selected anion-exchange durations. Estimated standard deviations are given in parentheses.

Sample	**Corresponding phase**	**Mass fraction (wt%)**	**Unit cell parameters**
5h-Sample	Cubic, Pm-3m	68.09%	a = 6.03841(27) Å<br>V = 220.175(30) Å³
	Tetragonal, I4/mcm	31.91%	a = 8.4813(5) Å,<br>c = 12.0984(4) Å<br>V = 870.27(11) Å³
10h-Sample	Cubic, Pm-3m	24.90%	a = 6.1524(9) Å<br>V = 232.88(10) Å³
	Tetragonal, I4/mcm	75.10%	a = 8.4788(20) Å,<br>c = 12.4950(4) Å<br>V = 898.3(4) Å³

Table S2. The EDS spectrum and full composition table of the $CH_3NH_3PbI_{3-x}Br_x$ films measured at different anion-exchange times. EDS measurement notes: Quantitative EDS of light elements (C, N) and halogens is affected by surface contamination, matrix effects, and topography-induced variations. Consequently, absolute atomic percentages are used only qualitatively; the I/(I+Br) ratio is adopted for compositional tracking because it offers better reproducibility by cancelling common systematic errors.

1 h:

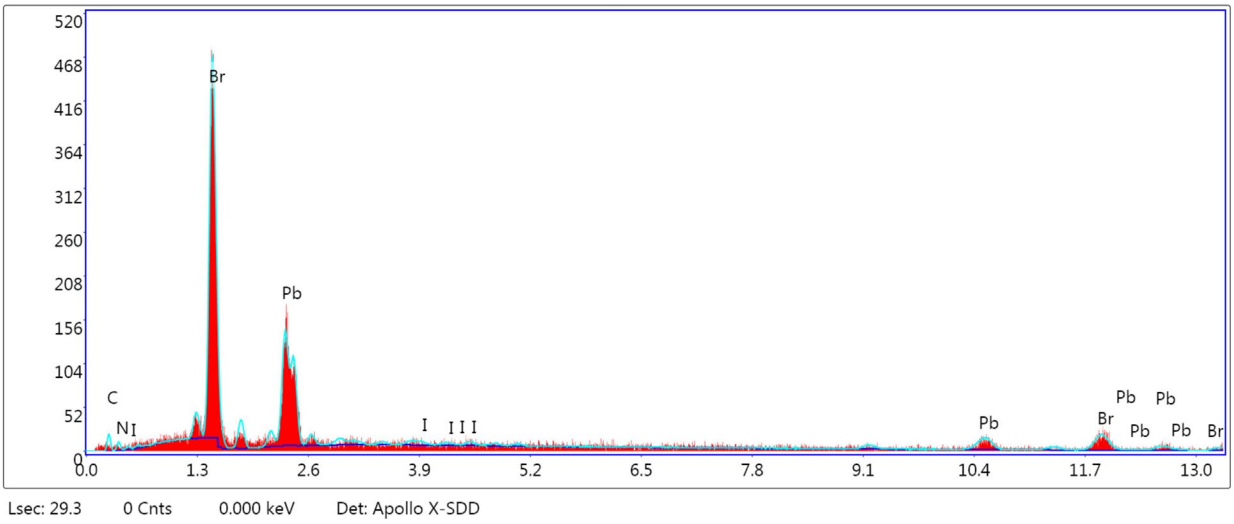

Element	Weight %	Atomic %	Net Int.	Error %	Kratio
C K	15.32	47.68	6.68	19.60	0.0288
N K	9.92	26.46	3.55	30.66	0.0132
BrL	42.97	20.09	208.68	4.43	0.3322
PbM	31.48	5.68	86.23	8.00	0.2185
I L	0.31	0.09	0.74	66.37	0.0023

2 h:

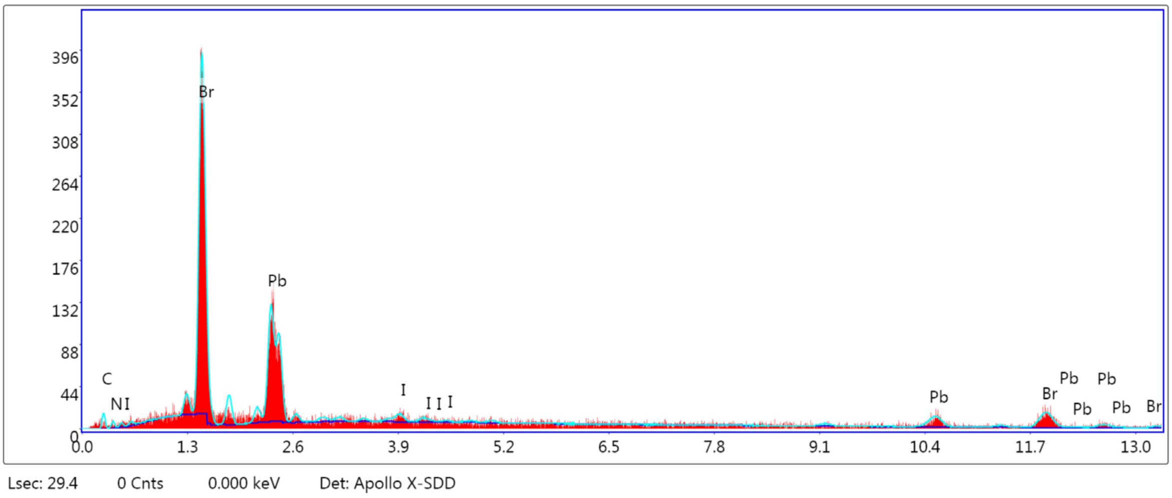

Element	Weight %	Atomic %	Net Int.	Error %	Kratio
C K	14.58	49.36	5.49	21.22	0.0280
N K	7.51	21.81	2.33	38.85	0.0103
BrL	42.38	21.56	169.73	5.22	0.3200
PbM	33.11	6.50	76.97	8.82	0.2311
I L	2.42	0.78	4.95	62.00	0.0182

3 h:

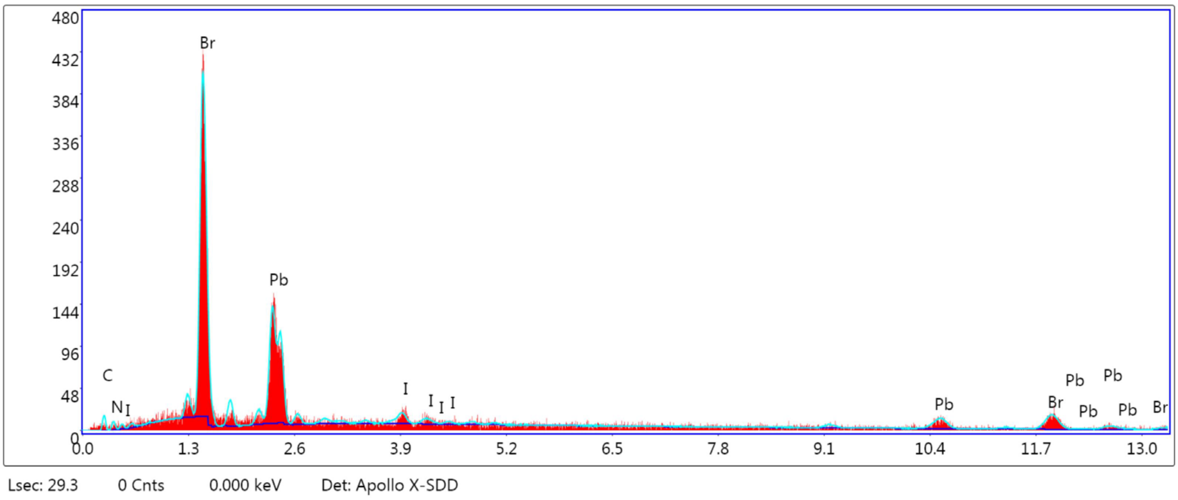

Element	Weight %	Atomic %	Net Int.	Error %	Kratio
C K	13.43	45.00	5.74	20.90	0.0263
N K	9.36	26.90	3.34	31.27	0.0133
BrL	41.00	20.65	179.78	5.22	0.3047
PbM	32.77	6.37	85.32	7.73	0.2302
I L	3.44	1.09	7.84	52.74	0.0259

4 h:

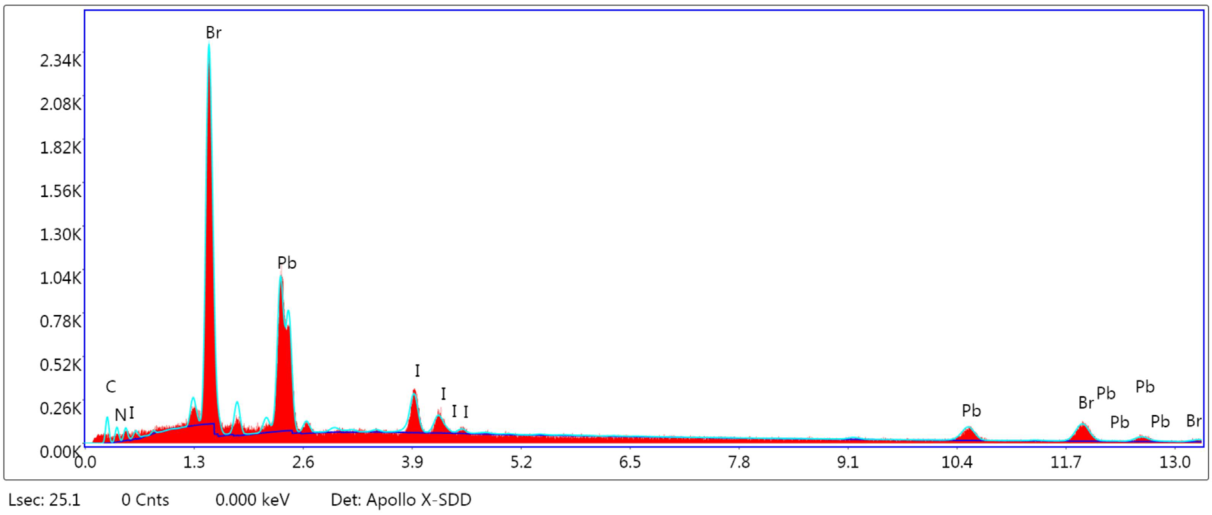

Element	Weight %	Atomic %	Net Int.	Error %	Kratio
C K	14.30	45.62	60.71	12.84	0.0314
N K	10.60	29.00	35.71	15.68	0.0160
BrL	34.89	16.73	1247.35	4.40	0.2387
PbM	29.77	5.50	705.16	3.75	0.2148
I L	10.43	3.15	213.98	10.92	0.0800

5 h:

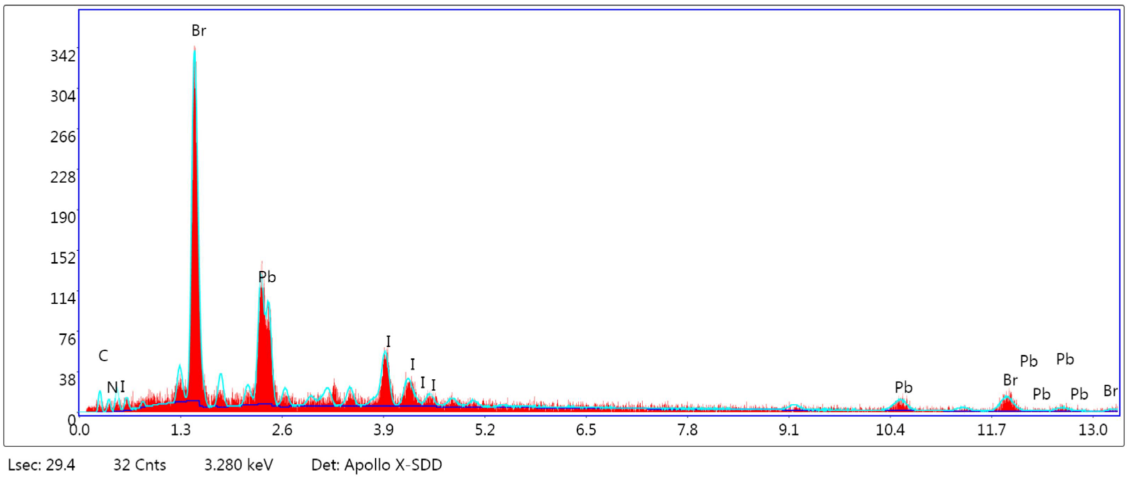

Element	Weight %	Atomic %	Net Int.	Error %	Kratio
C K	13.02	44.24	6.96	18.84	0.0286
N K	9.44	27.50	4.15	27.80	0.0147
BrL	35.58	18.18	155.34	6.04	0.2357
PbM	27.37	5.39	81.16	7.33	0.1960
I L	14.60	4.70	38.34	17.43	0.1136

7.5 h:

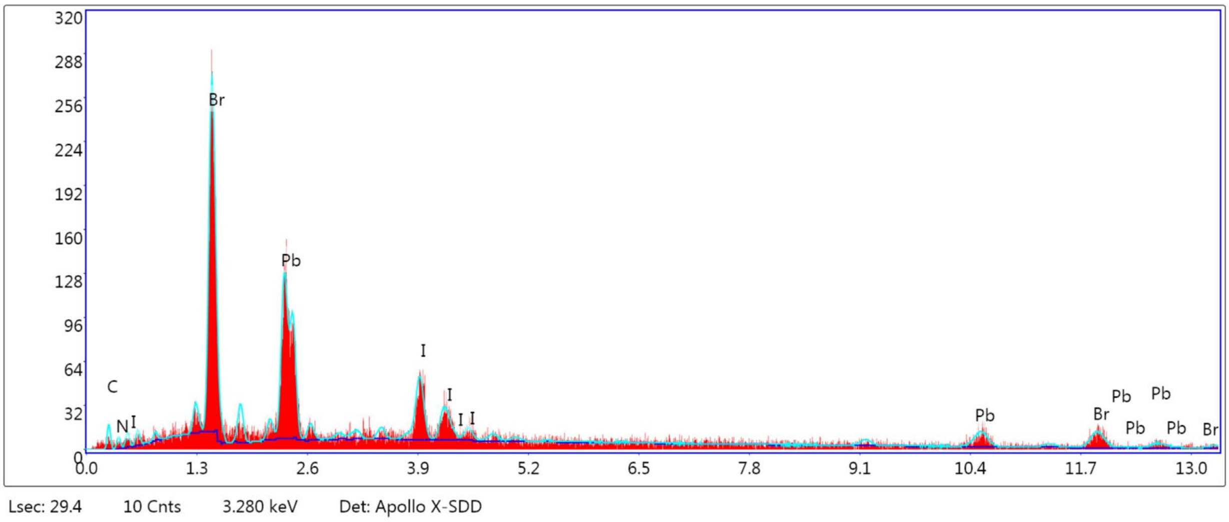

Element	Weight %	Atomic %	Net Int.	Error %	Kratio
C K	12.78	46.34	6.03	20.35	0.0298
N K	7.66	23.82	2.89	34.03	0.0123
BrL	32.91	17.93	117.10	7.03	0.2135
PbM	30.72	6.46	77.24	9.84	0.2242
I L	15.92	5.46	34.71	20.44	0.1236

10 h:

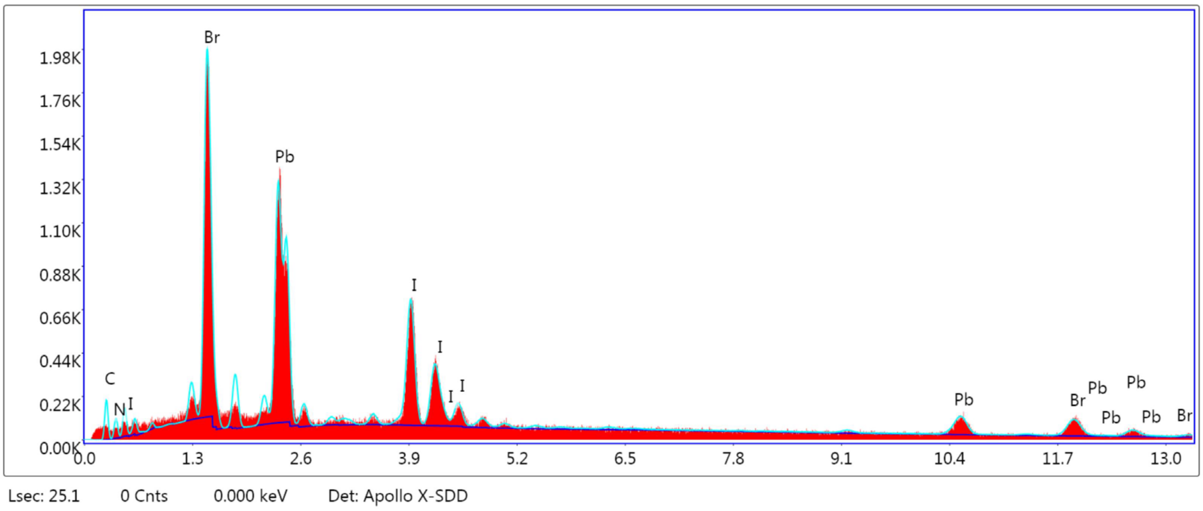

Element	Weight %	Atomic %	Net Int.	Error %	Kratio
C K	12.34	45.35	79.10	12.03	0.0330
N K	8.14	25.65	39.77	15.38	0.0144
BrL	26.65	14.72	1040.68	5.59	0.1607
PbM	30.41	6.48	932.22	3.45	0.2292
I L	22.46	7.81	585.51	6.15	0.1766

12.5 h:

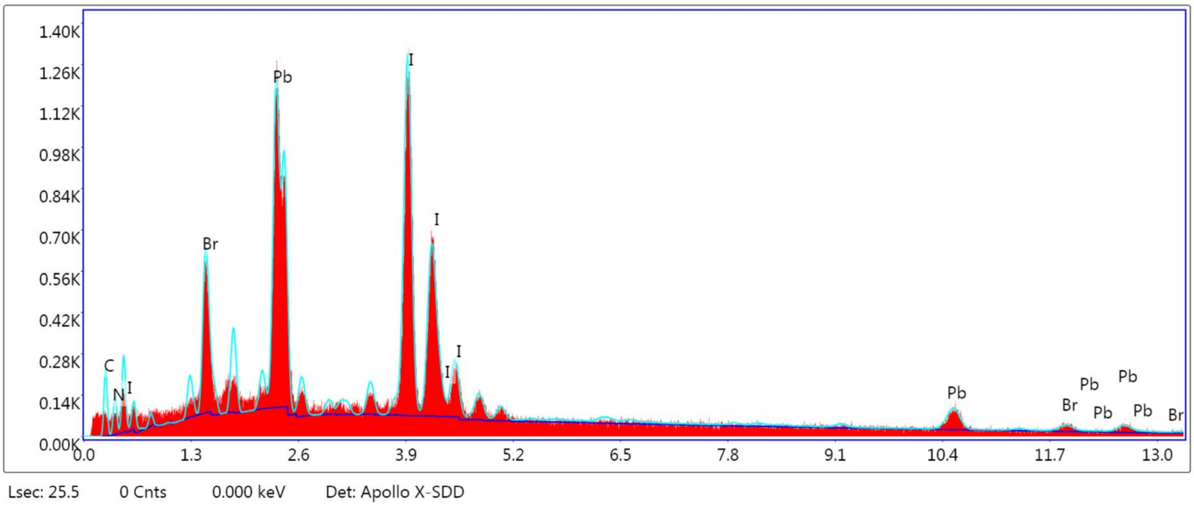

Element	Weight %	Atomic %	Net Int.	Error %	Kratio
C K	9.08	39.03	88.62	10.61	0.0399
N K	7.92	29.21	51.88	14.20	0.0202
BrL	9.96	6.44	293.20	8.02	0.0488
PbM	27.88	6.95	862.56	3.33	0.2285
I L	45.16	18.37	1149.23	3.69	0.3735

15 h:

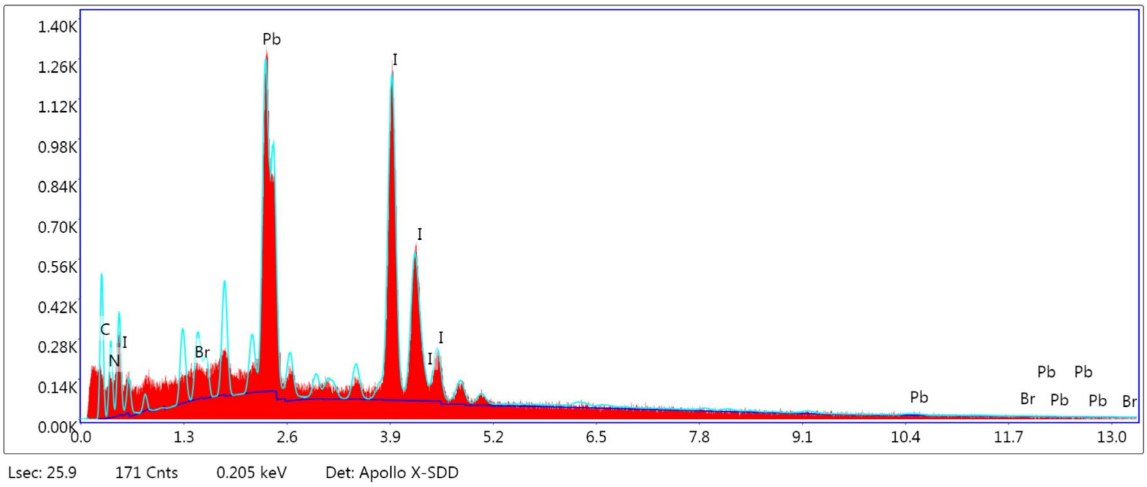

Element	Weight %	Atomic %	Net Int.	Error %	Kratio
C K	14.20	42.87	192.92	8.47	0.0799
N K	15.19	39.33	104.24	12.03	0.0373
BrL	3.29	1.49	105.50	8.26	0.0161
PbM	26.46	4.63	926.71	3.26	0.2258
I L	40.87	11.68	1107.28	3.44	0.3310
